# Editorial: Epidemiology and Genetics of Vestibular Disorders

**DOI:** 10.3389/fneur.2021.743379

**Published:** 2021-09-24

**Authors:** Jose A. Lopez-Escamez, Alan G. Cheng, Eva Grill, Tien-Chen Liu

**Affiliations:** ^1^Otology and Neurotology Group CTS495, Department of Genomic Medicine, GENYO - Centre for Genomics and Oncological Research - Pfizer/University of Granada/Junta de Andalucía, Parque Tecnologico de la Salud (PTS), Granada, Spain; ^2^Department of Otolaryngology, Instituto de Investigación Biosanitaria, ibs.GRANADA, Hospital Universitario Virgen de las Nieves, Granada, Spain; ^3^Division of Otolaryngology, Department of Surgery, Universidad de Granada, Granada, Spain; ^4^Sensorineural Pathology Programme, Centro de Investigacion Biomédica en Red en Enfermedades Raras (CIBERER), Madrid, Spain; ^5^Department of Otolaryngology - Head and Neck Surgery, Stanford University School of Medicine, Stanford, CA, United States; ^6^Institute for Medical Information Processing, Biometrics and Epidemiology, Ludwig-Maximilians-Universität München (LMU) Munich, Munich, Germany; ^7^German Center for Vertigo and Balance Disorders, University Hospital Munich, Ludwig-Maximilians-Universität München (LMU) Munich, Munich, Germany; ^8^Munich Centre of Health Sciences, Ludwig-Maximilians-Universität München (LMU) Munich, Munich, Germany; ^9^Department of Otolaryngology, National Taiwan University Hospital, Taipei, Taiwan

**Keywords:** Meniere disease, vestibular migraine, heritability, exome sequencing, RNA seq, familial aggregation

Vestibular disorders (VD) include a heterogeneous set of neuro-otological conditions. Peripheral and central VD such as vestibular migraine (VM) or Menière's disease (MD) are among the more frequently encountered disease entities, but there is also a large group of rare cerebellar disorders (1–3).

This issue of Frontiers in Neurology is dedicated to recent developments and new methodological findings in the epidemiology and genetics of VD. Of note, heritability has been largely ignored in VD as epidemiological evidence based on familial aggregation and twin studies are scarce (4, 5). Familial clustering suggests a genetic contribution in some VD, including VM, MD, and spinocerebellar and episodic ataxias (6, 7). Paz-Tamayo et al. show epidemiological evidence to support heritability in VM including familial aggregation and ethnic-specific differences in the occurrence of this condition.

However, a better characterization is needed to define syndromes and symptoms that overlap between individuals with a vestibular episodic syndrome and those with peripheral and central bilateral vestibular loss.

Precise history taking is the first and essential step for the diagnosis of vestibular disorders, and the systematic gathering of clinical information is particularly relevant in the primary care setting (Strobl et al.). The distribution of diagnoses in patients with VD is different in primary care and specialized neuro-otology clinics, and therefore they have different needs. Primary care professionals would benefit from training on maneuvers for repositioning otoliths, the diagnosis and treatment of different types of headaches, the identification of cardiovascular risk factors including orthostatic hypotension, and the appreciation of unwanted effects of some of the most commonly used drugs (Domínguez-Durán et al.) In an opinion paper, Maarsingh and van Vugt propose 10 practical vestibular tools for primary care physicians. In addition, machine learning techniques applied on large datasets have a huge potential to provide a decision support system for diagnosis and treatment in neuro-otology (Vivar et al.), including the classification of central and peripheral VD (8).

The assessment of disability is also a major issue in the elderly population and presbyvestibulopathy shows an important subjective perception of disability, particularly in women (Soto-Varela et al.). For this reason, co-morbidities should be carefully considered in patients with vestibular dysfunction (Malmström et al.), this approach being used to define clinical subgroups of patients (9, 10). Bilateral vestibulopathy (BVP) is a heterogeneous clinical condition characterized by a hypofunction of the vestibular nerves or labyrinths on both sides and quantitative assessment of the vestibulo-ocular reflex is needed to differentiate it from presbyvestibulopathy (11). In a retrospective study, Mancino-Moreira et al. classify patients into four clinical subgroups according to the symptoms: recurrent vertigo with BVP, rapidly progressive BVP, slowly progressive BVP, and slowly progressive BVP with ataxia.

Despite the huge progress in the definition and classification of vestibular disorders performed by the International Classification Committee, Dlugaiczyk et al. illustrate that there are still patients whose recurrent vestibular symptoms cannot be attributed to any of the recognized episodic vestibular syndromes, including MD (12), VM (13), benign paroxysmal positional vertigo (14), vestibular paroxysmia (15), orthostatic vertigo (16), or transient ischemic attacks (17). This category has been defined as recurrent vestibular symptoms not otherwise specified and it is composed of individuals with an incomplete phenotype not fulfilling the diagnostic criteria for MD or VM.

Research about the genetics of vestibular disorders in an emerging topic, including MD (18–20), and this volume offers some outstanding pictures that contribute to a better understanding of neurotological disorders. Gu et al. combine RNAseq and data mining to define potential MD genes in the stria vascularis. Shew et al. report microRNA profiles in the perilymph and serum of patients with MD that may serve as potential biomarkers of the condition. The diagnosis and prognosis of MD is likely to be improved by the presence of endolymphatic sac (ES) hypoplasia, under the hypothesis that ES hypoplasia critically predisposes the inner ear to develop bilateral MD (Bächinger et al.).

Rujescu et al. report an allelic variant conferring susceptibility to vestibular neuritis, indirect evidence for an involvement of Herpes simplex virus in this condition. Mei et al. highlight the role of genetic sequencing to develop personalized medicine in VD. As an example, Oh et al. report the *TRPM7* gene in a Korean family with four affected individuals with vestibular migraine as the first candidate gene for familial vestibular migraine by exome sequencing. Choi et al. also report MD-like symptoms in the 22q11.2 deletion syndrome, targeting the *TBX1* gene. Moreover, epigenetic regulation by circular RNAs may explain susceptibility for intracranial aneurysms rupture (Huang et al.).

This is only the beginning. Genetic research in VD is still in its infancy. The development of cellular and animal models of vestibular disorders is needed to carry out functional validation of candidate genes obtained in human studies (21, 22). Gene replacement therapy can successfully repair auditory and vestibular hair cells and preserve organ function in genetic mouse models (23).

## Author Contributions

JL-E wrote the original draft, assembled and incorporated comments from the co-authors, and crafted the final draft. All co-authors contributed to manuscript review and revision.

## Funding

JL-E received research support from the Instituto de Salud Carlos III, European Regional Funds (Grant PI20/1126), Andalusian Family & Health Department (Grant PI027/2020), and the European Union (Horizon 2020, Grant Agreement 848261).

## Conflict of Interest

The authors declare that the research was conducted in the absence of any commercial or financial relationships that could be construed as a potential conflict of interest. The handling editor declared shared affiliation with one of the authors, EG, at time of review.

## Publisher's Note

All claims expressed in this article are solely those of the authors and do not necessarily represent those of their affiliated organizations, or those of the publisher, the editors and the reviewers. Any product that may be evaluated in this article, or claim that may be made by its manufacturer, is not guaranteed or endorsed by the publisher.
